# CRISPR-assisted transcription activation by phase-separation proteins

**DOI:** 10.1093/procel/pwad013

**Published:** 2023-03-11

**Authors:** Jiaqi Liu, Yuxi Chen, Baoting Nong, Xiao Luo, Kaixin Cui, Zhan Li, Pengfei Zhang, Wenqiong Tan, Yue Yang, Wenbin Ma, Puping Liang, Zhou Songyang

**Affiliations:** State Key Laboratory of Biocontrol, MOE Key Laboratory of Gene Function and Regulation and Guangzhou Key Laboratory of Healthy Aging Research, School of Life Sciences, Sun Yat-sen University, Guangzhou 510275, China; State Key Laboratory of Biocontrol, MOE Key Laboratory of Gene Function and Regulation and Guangzhou Key Laboratory of Healthy Aging Research, School of Life Sciences, Sun Yat-sen University, Guangzhou 510275, China; State Key Laboratory of Biocontrol, MOE Key Laboratory of Gene Function and Regulation and Guangzhou Key Laboratory of Healthy Aging Research, School of Life Sciences, Sun Yat-sen University, Guangzhou 510275, China; Sun Yat-sen Memorial Hospital, Sun Yat-sen University, Guangzhou 510275, China; State Key Laboratory of Biocontrol, MOE Key Laboratory of Gene Function and Regulation and Guangzhou Key Laboratory of Healthy Aging Research, School of Life Sciences, Sun Yat-sen University, Guangzhou 510275, China; State Key Laboratory of Biocontrol, MOE Key Laboratory of Gene Function and Regulation and Guangzhou Key Laboratory of Healthy Aging Research, School of Life Sciences, Sun Yat-sen University, Guangzhou 510275, China; State Key Laboratory of Biocontrol, MOE Key Laboratory of Gene Function and Regulation and Guangzhou Key Laboratory of Healthy Aging Research, School of Life Sciences, Sun Yat-sen University, Guangzhou 510275, China; State Key Laboratory of Biocontrol, MOE Key Laboratory of Gene Function and Regulation and Guangzhou Key Laboratory of Healthy Aging Research, School of Life Sciences, Sun Yat-sen University, Guangzhou 510275, China; Lumiere Therapeutics, Suzhou 215000, China; State Key Laboratory of Biocontrol, MOE Key Laboratory of Gene Function and Regulation and Guangzhou Key Laboratory of Healthy Aging Research, School of Life Sciences, Sun Yat-sen University, Guangzhou 510275, China; State Key Laboratory of Biocontrol, MOE Key Laboratory of Gene Function and Regulation and Guangzhou Key Laboratory of Healthy Aging Research, School of Life Sciences, Sun Yat-sen University, Guangzhou 510275, China; State Key Laboratory of Biocontrol, MOE Key Laboratory of Gene Function and Regulation and Guangzhou Key Laboratory of Healthy Aging Research, School of Life Sciences, Sun Yat-sen University, Guangzhou 510275, China; State Key Laboratory of Biocontrol, MOE Key Laboratory of Gene Function and Regulation and Guangzhou Key Laboratory of Healthy Aging Research, School of Life Sciences, Sun Yat-sen University, Guangzhou 510275, China; Sun Yat-sen Memorial Hospital, Sun Yat-sen University, Guangzhou 510275, China

**Keywords:** CRISPR, transcriptional activation, phase-separation proteins

## Abstract

The clustered regularly interspaced short palindromic repeats (CRISPR)-Cas9 system has been widely used for genome engineering and transcriptional regulation in many different organisms. Current CRISPR-activation (CRISPRa) platforms often require multiple components because of inefficient transcriptional activation. Here, we fused different phase-separation proteins to dCas9-VPR (dCas9-VP64-P65-RTA) and observed robust increases in transcriptional activation efficiency. Notably, human NUP98 (nucleoporin 98) and FUS (fused in sarcoma) IDR domains were best at enhancing dCas9-VPR activity, with dCas9-VPR-FUS IDR (VPRF) outperforming the other CRISPRa systems tested in this study in both activation efficiency and system simplicity. dCas9-VPRF overcomes the target strand bias and widens gRNA designing windows without affecting the off-target effect of dCas9-VPR. These findings demonstrate the feasibility of using phase-separation proteins to assist in the regulation of gene expression and support the broad appeal of the dCas9-VPRF system in basic and clinical applications.

## Introduction

Bacteria and archaea utilize CRISPR and CRISPR-associated proteins (CRISPR-Cas) as part of their adaptive immune systems against phage and other foreign genetic elements (Koonin et al., 2017; Hille et al., 2018; Makarova et al., 2020). The class 2 type II effector Cas9 and type V effector Cas12 in particular have revolutionized both basic and clinical research in cultured cells and whole animals (Knott and Doudna, 2018; Uddin et al., 2020). For instance, Cas9 has been used in base editing (CBEs and ABEs) (Komor et al., 2016; Gaudelli et al., 2017), imaging of specific DNA loci (Chen et al., 2013), transcriptional regulation (Gilbert et al., 2013; Qi et al., 2013), and epigenome editing (Hilton et al., 2015) in human cells. Gene expression regulation using nuclease-inactivated Cas9 promises safer therapies than active Cas9, as cleavage by the latter will induce double-strand breaks that can result in large fragment deletions and chromothripsis (Adikusuma et al., 2018; Cullot et al., 2019).

CRISPRa (CRISPR activation) systems are among the most widely used endogenous gene expression activators, where nuclease-dead Cas proteins are fused to transcription factors (TFs) (e.g., VP64) (Maeder et al., 2013; Perez-Pinera et al., 2013; Chavez et al., 2015) or DNA/histone modification enzymes (e.g., TET1 or the catalytic core domain of p300) (Hilton et al., 2015; Liu et al., 2016). For fundamental investigations, CRISPRa systems have been used for DNA *cis*-regulatory elements annotating (Liu et al., 2018), gene function analysis (Savell et al., 2019), cell signaling analysis (Baeumler et al., 2017; Kipniss et al., 2017), and so on. For pre-clinical therapeutic studies, CRISPRa systems have been used in mouse models for disease treatment, and these diseases were caused by severe haploinsufficiency of specific genes or complete loss of functional genes which could be compensated by activating other genes. For example, activating the *Sim1* gene in mice bearing obesity (*Sim1* heterozygous knockout mice) to rescue the obesity phenotype (Matharu et al., 2019), up-regulating the *Utrn* gene in mice (*Dmd* gene loss-of-function mutant mice) to treat muscular dystrophy (Liao et al., 2017), etc. Low efficiency remains a major roadblock for CRISPRa systems in basic and clinical applications and much effort has been devoted to combating this issue (Konermann et al., 2015; Zhou et al., 2018). In principle, the efficiency of gene activation can be enhanced by increasing the local concentration of TFs and co-activators. For example, the gRNA scaffold in the SAM system was fused to the MS2 hairpin which led to more TFs being recruited through MS2–MCP interaction (Konermann et al., 2015), dCas9 in the SUN and SPH systems were fused to 10× GCN4 to recruit more TFs through GCN4–scFv interaction (Tanenbaum et al., 2014; Zhou et al., 2018). We have also shown previously that local TF concentration could be increased by using multiple copies of Csy3-VPR in the Cascade-VPR complex (Chen et al., 2020). It should be noted that the above methods all raise local TF concentration through increasing TF binding valency at target sites, which necessitates the inclusion of multiple components that can severely interfere with the construction of efficient gene therapy vectors (Liao et al., 2017; Zhou et al., 2018).

Recent studies have revealed that biomolecules can form compartmentalized membrane-less organelles via liquid-liquid phase separation in cells, in which biochemical reactions can occur efficiently (Banani et al., 2017; Spannl et al., 2019). For example, transcription may be carried out and regulated in membrane-less organelles such as nucleoli (rRNA transcription and ribosome biogenesis) (Boisvert et al., 2007; Brangwynne et al., 2011; Feric et al., 2016), histone locus bodies (histone mRNA biogenesis) (Nizami et al., 2010; Tatomer et al., 2016; Duronio and Marzluff, 2017), PML bodies (transcriptional regulation) (Zhong et al., 2000), and nuclear stress bodies (control of transcription and splicing activities) (Biamonti and Vourc’h, 2010; Niemelä et al., 2019). Transcriptional regulation may be executed in large as well as small assembly condensates (Boija et al., 2018; Cho et al., 2018; Chong et al., 2018; Sabari et al., 2018), including the mediator-RNA polymerase II clusters (Cho et al., 2018) and the coactivator condensation at super-enhancer clusters (Sabari et al., 2018). Transcriptional condensates are enriched in phase-separation associated proteins that contain intrinsically disordered regions (IDRs), such as members of the FET protein family FUS (fused in sarcoma) (also known as TLS), EWS (also known as the Ewing sarcoma RNA-binding protein, EWSR1), and TAF15 (TATA-box binding protein associated factor 15) (Schwartz et al., 2015), and the NPC (nuclear pore complex) proteins NUP98 (Nucleoporin 98), and NUP50 (Nucleoporin 50) (Lin and Hoelz, 2019), etc. IDR-rich proteins can bind themselves (oligomerization) (Shin et al., 2017; Zhou et al., 2020; Carter et al., 2021), DNA or RNA (Feric et al., 2016; Mitrea et al., 2016; Smith et al., 2016), and proteins (Tatomer et al., 2016; Boija et al., 2018; Chong et al., 2018; Sabari et al., 2018) through multivalent interactions to promote phase separation and gene transcription (Schwartz et al., 2015). In addition, the IDR-rich EWS fusion protein and the TF FLI1 (Friend leukemia integration 1 TF) can bind repetitive DNA sequences and induce phase separation to upregulate gene expression in cancer cells (Chong et al., 2018).

We reasoned that by harnessing the multivalent binding potential of IDR-rich proteins such as NUP98 and FUS, we might be able to increase the activity of CRISPRa and a similar strategy has been used for CRISPR-assisted genomic imaging (Lyu et al., 2022). We, therefore, screened a collection of IDR-rich proteins and developed two robust CRISPRa systems that we named dCas9-VPRN and dCas9-VPRF. Here, the IDR sequence of NUP98 (a.a. 1–515) or FUS (a.a. 1–212) was fused to dCas9-VPR., which led to drastically enhanced transcriptional activation without significantly increasing the system’s overall complexity. We believe that both dCas9-VPRN and dCas9-VPRF hold great promise for both basic research (e.g., CRISPRa-based screening) (Gilbert et al., 2014; Brezgin et al., 2019) and clinic therapy that seeks to activate functionally equivalent genes of diseases relevant mutated genes to treat diseases (e.g., *HBG* activation for β-thalassemia patients) (Makis et al., 2001; Becirovic, 2022; Riedmayr et al., 2022).

## Results

### A systemic screen for efficient CRISPRa systems that utilize IDR-rich proteins

**Figure 1.**
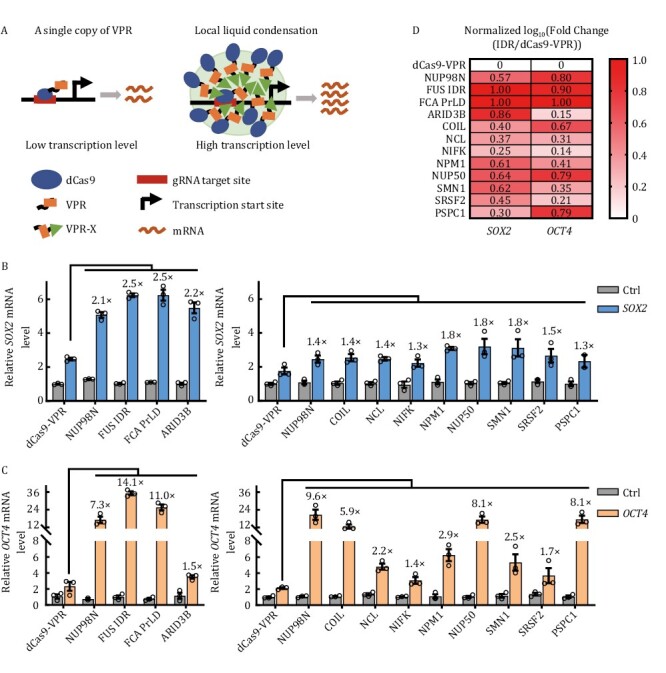
**Screening for efficient CRISPRa systems by fusing IDR-rich proteins to dCas9-VPR**. (A) Generally, only a limited amount of dCas9-VPR can bind to the target DNA sequence, leading to often inadequate transcriptional activation. Fusing IDR-rich phase-separation proteins (denoted as X) to dCas9-VPR may lead to local liquid condensation thereby increasing the binding valency of dCas9-VPR, resulting in enhanced transcriptional activation. (B and C) HEK293T cells transiently expressing the indicated dCas9-VPR fusion proteins (C-terminal fusion) together with a gRNA-targeting either *SOX2* (−185 bp) (B) or *OCT4* (−283 bp) were harvested for RT-qPCR analysis of mRNA expression 48 h after transfection. Ctrl, no gRNA control. The activation fold change of dCas9-VPR-X fusions over dCas9-VPR was indicated above the column. The results are presented as mean ± S.E.M. and represent three biological repeats. (D) Data from (B) and (C) were processed to obtain the ratio of fold changes in gene expression activity by different dCas9-VPR/IDR fusion proteins over dCas9-VPR alone. For each target gene, the values of log_10_(fold change) were normalized with a minimum value of zero and a maximum value of one by GraphPad Prism and presented in the heat map.

To test our hypothesis that including phase-separation associated proteins can increase the binding valency of dCas9-VPR-based CRISPRa thereby enhancing its activity (Fig. 1A), we generated a series of dCas9-VPR fusion proteins with full-length phase-separation associated proteins or their IDR-rich regions (Fig. S1). These include nucleoporin 98 (NUP98) (a.a. 1–514), FUS (a.a. 1–212) (Murakami et al., 2015) from human, the flowering time control protein FCA (a.a. 349–747) from *Arabidopsis* (Fang et al., 2019), AT-rich interactive domain-containing protein 3B (ARID3B, a.a. 1–567) from mouse (Wilsker et al., 2005), the cajal body component COIL (a.a. 1–576) (Machyna et al., 2015), nucleolin (NCL) (a.a. 1–710) (Jia et al., 2017), NIFK (nucleolar protein interacting with the FHA domain of MKI67) (a.a. 1–293) (Takagi et al., 2001), nucleophosmin 1 (NPM1) (a.a. 1–294) (Falini et al., 2007; Box et al., 2016), nucleoporin 50 (NUP50) (a.a. 1–468) (Lindsay et al., 2002; Moore, 2003), SRSF2 (splicing factor, arginine/serine-rich 2) (a.a. 1–221) (Kielkopf, 2018), survival of motor neuron 1 (SMN1) (a.a.1–294) (Singh et al., 2017), and paraspeckle protein component 1 (PSPC1) (a.a. 1–523) from human (Passon et al., 2012; Knott et al., 2016). We compared the ability of these fusion proteins to activate *SOX2*, *OCT4*, and *IL1RN*. Compared to dCas9-VPR alone, the addition of phase-separation proteins clearly enhanced the ability of dCas9-VPR to activate target gene expression (Figs. 1B, 1C, and S2a), with FUS IDR and FCA PrLD, and to a lesser extent NUP98N, NUP50, being particularly strong (Figs. 1D and S2b). These observations are consistent with the published reports that the NUP98 (NUP98-HOXA9) and FUS (FUS-CHOP) fusion proteins could robustly promote transcriptional activation (Crozat et al., 1993; Rabbitts et al., 1993; Borrow et al., 1996; Nakamura et al., 1996). Given the potential of CRISPRa systems in basic and clinical research, we decided to look more closely at how NUP98/FUS fusion with dCas9-VPR was able to elevate CRISPRa activity.

### NUP98N fusion with dCas9-VPR enables the formation of phase-separated condensates

**Figure 2.**
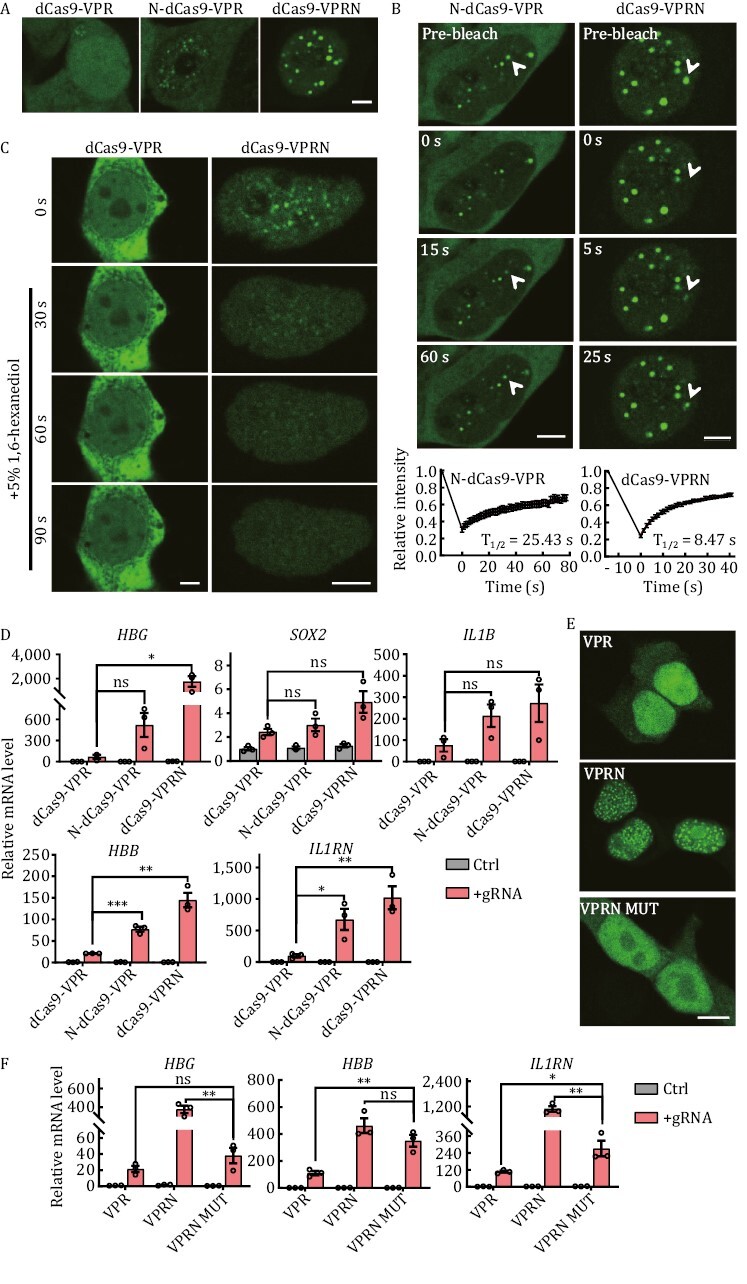
**NUP98 fusion with dCas9-VPR leads to condensate formation and higher CRISPRa activity**. (A) Live-cell imaging of HEK293T cells transiently expressing GFP-tagged N- or C-terminal fusion proteins of NUP98N and dCas9-VPR. N-dCas9-VPR, N-terminal fusion. dCas9-VPRN, NUP98N fused to the C-terminus of dCas9-VPR. GFP-tagged dCas9-VPR alone served as controls. Scale bar, 5 μm. (B) Fluorescence recovery after photobleaching (FRAP) analysis of cells from (A). Top, representative images of cells at the indicated time points following photobleaching. White arrowheads indicate photobleached condensates that were in recovery. Scale bar, 5 μm. Bottom, recovery curves of the condensates were plotted mean ± S.E.M. Three independent experiments were performed with ~15 cells/experiment. All cells had at least one photobleached condensate. The recovery curves were fitted to determine the half-life (*T*_1/2_). (C) HeLa cells transiently expressing GFP-tagged C-terminal fusion proteins of NUP98N and dCas9-VPR (dCas9-VPRN) were treated with 5% 1,6-hexanediol 24 h after transfection and imaged at the indicated time points following treatment. GFP-tagged dCas9-VPR alone served as controls. Scale bar, 5 μm. (D) HEK293T cells transiently co-expressing GFP-tagged N-dCas9-VPR or dCas9-VPRN with gRNAs targeting the indicated genes were harvested 48 h after transfection for RT-qPCR analysis. dCas9-VPR alone served as a control. Ctrl, no gRNA. Data from three biological repeats were graphed as mean ± S.E.M. Statistical significance was calculated using the two-tailed *t*-test. ns, not significant; ^*^P < 0.05; ^**^P < 0.01; ^***^P < 0.001. (E) Fixed cell imaging of HEK293T cells transiently expressing GFP-tagged C-terminal fusion proteins of NUP98N or NUP98N MUT and dCas9-VPR. VPR, dCas9-VPR. VPRN, dCas9-VPR-NUP98N. VPRN MUT, dCas9-VPR-NUP98N MUT. NUP98N MUT, Phe to Ser amino acid mutant of NUP98N, is a phase-separation-deficient protein. Scale bar, 10 μm. (F) HEK293T cells transiently co-expressing GFP-tagged dCas9-VPR fusion proteins and gRNAs targeting the indicated genes were harvested 48 h after transfection for RT-qPCR analysis. VPR, dCas9-VPR. VPRN, dCas9-VPR-NUP98N. VPRN MUT, dCas9-VPR-NUP98N MUT. Ctrl: no gRNA. Data from three biological repeats were graphed as mean ± S.E.M. Statistical significance was calculated using the two-tailed *t*-test. ^*^P < 0.05; ^**^P < 0.01; ^***^P < 0.001.

NUP98 is a component of the nuclear pore complex (NPC) and can activate gene expression in *Drosophila* (Capelson et al., 2010; Kalverda et al., 2010). Recent studies also revealed that fusions between IDR-rich NUP98 and TFs or epigenetic modifiers (such as driver genes in pediatric leukemias—homeobox protein Hox-A9 and lysine-specific demethylase 5A), induced aberrant transcriptional activity by phase separation, leading to cancer (Ahn et al., 2021; Chandra et al., 2022; Terlecki-Zaniewicz et al., 2021). Its N-terminal domain (NUP98N) is capable of forming phase-separation condensates *in vitro* and in human cells (Terlecki-Zaniewicz et al., 2021; Xu et al., 2021a). We fused the N-terminal domain of NUP98, which consists of two FG (phenylalanine-glycine) repeats separated by the GLEBS (Gle2-binding site) sequence (Gough et al., 2011; Michmerhuizen et al., 2020), to either the N- or C-terminus of GFP-tagged dCas9-VPR (N-dCas9-VPR and dCas9-VPRN) and carried out photobleaching and live-cell imaging in HEK293T cells (Fig. 2A and 2B). Both NUP98N fusion proteins were able to form foci in the transfected cells, indicating the formation of phase-separated condensates, which were absent in cells expressing dCas9-VPR alone. The recovery half-times (*T*_1/2_) following photobleaching for N- and C-terminal fusion were 25.43 s and 8.47 s respectively (Fig. 2B), suggesting that C-terminal tagging of dCas9-VPR with NUP98N (dCas9-VPRN) may be preferable. In HeLa cells expressing the fusion proteins, similar condensates were also observed, which disappeared within 30 s after the addition of 5% 1,6-hexanediol (1,6-HD) (Fig. 2C), consistent with the previous observations that the nuclear puncta formed by GFP-tagged NUP98N fusion protein (NUP98-KDM5A) were rapidly resolved upon 1,6-HD treatment (Terlecki-Zaniewicz et al., 2021). These results support the notion that NUP98N was able to drive the dCas9-VPR/NUP98N fusion proteins to form phase-separated condensates. In the presence of gene-targeting gRNAs, the dCas9-VPR/NUP98N fusion proteins consistently activated endogenous gene expression more effectively than dCas9-VPR alone at all the loci tested, as much as ~20× higher in the case of dCas9-VPRN for *HBG* (Fig. 2D). This elevation in mRNA levels correlated with elevated protein expression as well (Fig. S3a). Importantly, the dCas9/NUP98N fusion protein without VPR was incapable of activating transcription (Fig. S3b), whereas the phase-separation deficient dCas9-VPRN MUT (the Phe residues in NUP98N FG repeats were mutated to Ser residues and such mutation did not affect the protein stability but abolished the droplet formation by NUP98N MUT fusion protein (Ahn et al., 2021)) could not promote transcriptional activation as high as dCas9-VPRN (Fig. 2E and 2F). In conclusion, the results indicated that transcriptional activator increase at the target site was key to dCas9-VPR/NUP98N fusions outperforming dCas9-VPR, but other elements also accounted for the higher CRISPRa activity, including the interacting proteome of NUP98N, protein stability, and expression level of the fusion protein, etc.

### NUP98N fusion with dCas9-VPR enables robust transcriptional activation with less restricted target site selection

**Figure 3.**
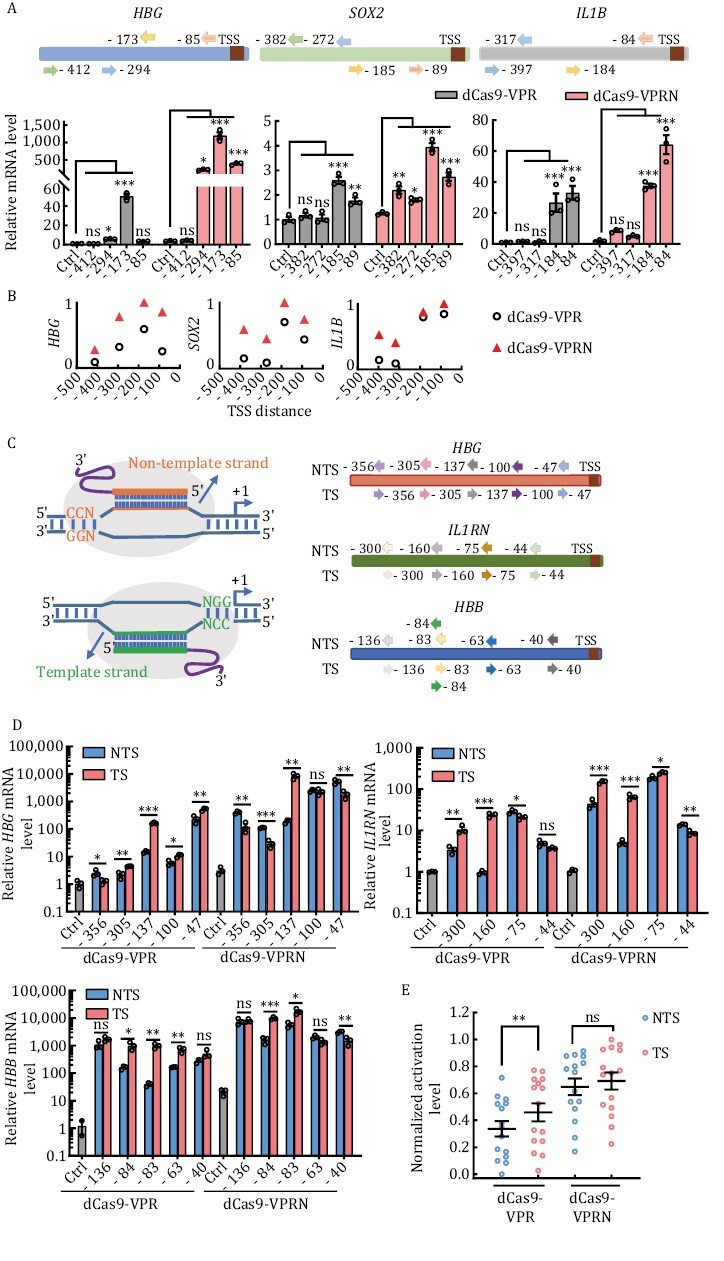
**The dCas9-VPRN system exhibits higher activity with less target site selection restriction**. (A) HEK293T cells were transfected with dCas9-VPR or dCas9-VPRN expression plasmids together with gRNAs targeting different sequences upstream of the transcription start sites (TSS) in the *HBG, SOX2,* or *IL1B* locus. Cells were collected 48 h post-transfection for RT-qPCR analysis. Top, gRNA-targeting sites at each locus (arrows represent the specific location). Bottom, results from three biological repeats were graphed as mean ± S.E.M. Statistical significance was calculated using one-way ANOVA. ns, not significant; ^*^P < 0.05; ^**^P < 0.01; ^***^P < 0.001. Ctrl, no gRNA control. (B) The mean fold activation of each target site was calculated based on results from (A) and the values of log_10_(mean) across different target regions of each gene locus were normalized with a minimum value of zero and a maximum value of one and plotted using GraphPad Prism. Each open circle or triangle represents the value of normalized log_10_(mean) for a target site. (C) Left, gRNA design targeting either the template (TS) or non-template (NTS) strand. Right, the specific regions targeted by the gRNAs at the indicated loci. (D) HEK293T cells transiently co-expressing dCas9-VPR or dCas9-VPRN together with gRNAs from (C) were assayed as described in (A). Results from three biological repeats are presented as mean ± S.E.M. Statistical significance was calculated using the two-tailed *t*-test. ns, not significant; ^*^P < 0.05; ^**^P < 0.01; ^***^P < 0.001. (E) The mean fold activation of each target site was calculated based on results from (D) and the values of log_10_(mean) of all target sites in dCas9-VPR or dCas9-VPRN were normalized with a minimum value of zero and a maximum value of one respectively and plotted as mean ± S.E.M. using GraphPad Prism. Statistical significance was calculated using a two-tailed *t*-test. ns, not significant; ^**^P < 0.01. Each open circle represents the value of normalized log_10_(mean) for a target site. Each center line indicates the mean.

Previous studies have demonstrated that the CRISPRa systems are efficient when targeting the region ~0 to 400 bp upstream of the transcription start site (TSS) in human cells, and they appear the most efficient at targeting within 0–200 bp with lower activity beyond 200 bp (Gilbert et al., 2014; Konermann et al., 2015; Heman-Ackah et al., 2016; Martella et al., 2019). Furthermore, it has been reported that gRNAs targeting the non-coding strand (also called template strand here) perform better than those targeting the coding strand (also called non-template strand here) in plants but the opposite in *Drosophila* (Mao et al., 2020; Pan et al., 2021). We next designed a series of gRNAs targeting different regions upstream of the TSS of several genes. Given that the C-terminal fusion of dCas9-VPR with NUP98N (dCas9-VPRN) was more efficient for phase separation and transcriptional activation, dCas9-VPRN was used for these studies. Once again, dCas9-VPRN surpassed dCas9-VPR in transcriptional activation at all the loci we tested (Figs. 3A and S4a). As reported previously, the dCas9-VPR CRISPRa system was the most effective when targeting regions ~0 to 200 bp upstream of TSS and the activity decreased quickly targeting beyond ~200 bp (Figs. 3B and S4b). In comparison, dCas9-VPRN robustly upregulated gene expression at target sites ~300 bp upstream of the TSS (Figs. 3B and S4b). A broader targeting window should be particularly useful for loci with limited gRNA choices in TSS-proximal regions and/or less responsive to dCas9-VPR alone.

To examine whether dCas9-VPRN also exhibits target strand preference in human cells, we designed pairs of gRNAs that target the same site on the non-template (NTS) vs. template (TS) strand and contain the same PAM and complementary sequences (Fig. 3C). Consistent with the data above, dCas9-VPRN showed higher efficiency than dCas9-VPR regardless of the strand being targeted (Figs. 3D and S4c). Although dCas9-VPR performed better when targeting the template strand in the target sites tested in this paper, dCas9-VPRN worked well targeting both strands with only no preference for TS (Fig. 3E). But further studies are required to confirm the result. Collectively, our results found dCas9-VPRN to exhibit higher activity, a wider target window, and less strand preference compared to dCas9-VPR.

### dCas9-VPRF is a more compact and efficient CRISPRa system

Several modified CRISPRa systems that utilize RNA-binding proteins or antibodies have been developed to increase the local concentration of TFs or coactivators (Fig. S5) (Tanenbaum et al., 2014; Konermann et al., 2015; Zhou et al., 2018). For instance, synergistic activation mediator (SAM) consists of the dCas9-VP64 fusion protein, an MS2-containing chimeric gRNA, and the MCP-P65-HSF1 fusion protein. Similarly, both SUN (SunTag) and SPH (SunTag-p65-HSF1) systems require a gRNA, the dCas9-10×GCN4 fusion protein, and the scFv-TF (scFv-VP64-P65-RTA and scFv-p65-HSF1 respectively) fusion protein. Such systems are complicated and require three independent expression modules (dCas9-VP64/dCas9-10×GCN4, MCP-P65-HSF1/scFv-VP64-P65-RTA/scFv-p65-HSF1, and gRNA), which may enlarge the systems and lead to low rAAV (recombination adeno-associated virus) packaging efficiency (Liao et al., 2017; Zhou et al., 2018; Riedmayr et al., 2022), given the capacity limit of rAAV (4.7 kb) (Wu et al., 2010). In comparison, our two-component dCas9-VPRN CRISPRa system (the dCas9-VPR-NUP98N fusion and a gRNA) offers clear advantages for delivery into cells. NUP98N, however, is still quite big with 514 amino acids. Further truncation of this protein resulted in a reduction of activation efficiency. Considering the potential spatial constraints for factor binding at target sites, smaller and more compact CRISPRa systems should also be more effective at increasing binding valence and further reducing the overall size of the system.

**Figure 4.**
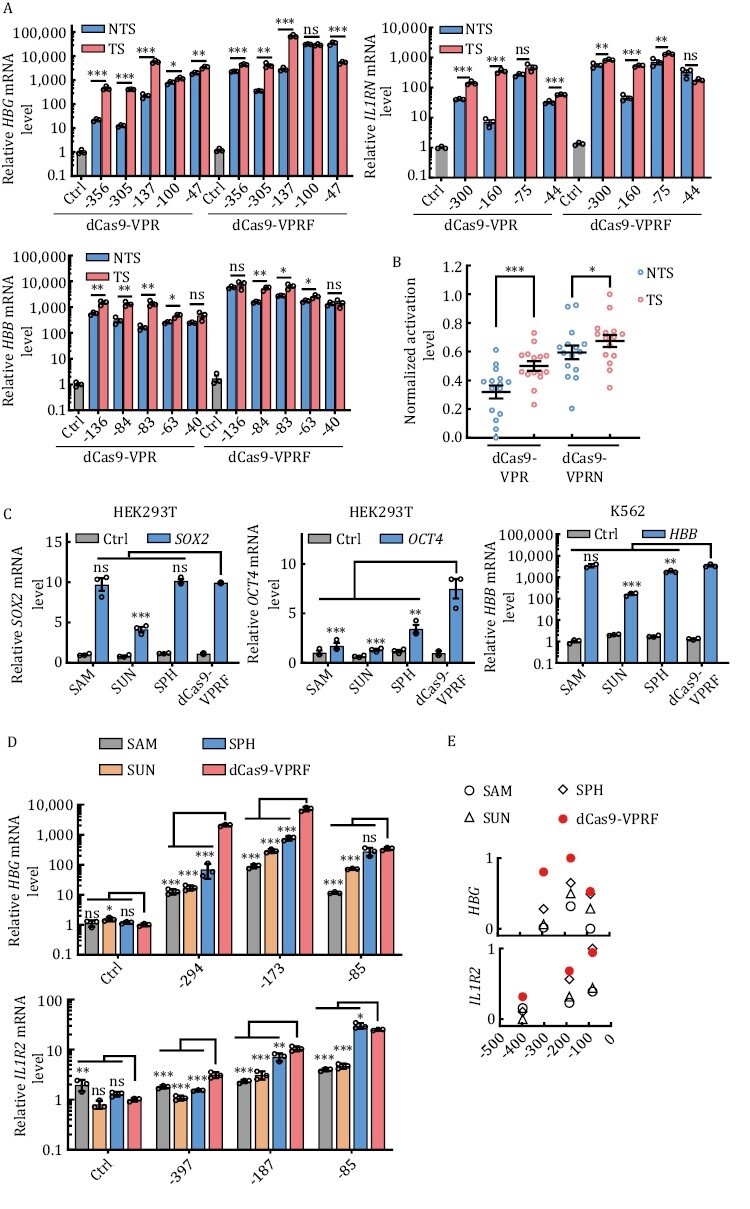
**The dCas9-VPRF system is efficient and less restrictive than other modified CRISPRa systems**. (A) HEK293T cells transiently expressing dCas9-VPR or dCas9-VPRF together with gRNAs targeting the regions indicated in Fig. 3C were harvested 48 h post-transfection for RT-qPCR analysis. The data were graphed as mean ± S.E.M. and represent three biological repeats. Statistical significance was calculated using the two-tailed *t*-test. ns, not significant. ^*^P < 0.05; ^**^P < 0.01; ^***^P < 0.001. NTS, non-template strand. TS, template strand. (B) The mean fold activation of each target site was calculated based on results from (A) and the values of log_10_(mean) of all target sites in three genes (*HBG*, *IL1RN*, *HBB*) from dCas9-VPR or dCas9-VPRF were normalized with a minimum value of zero and a maximum value of one individually and plotted as mean ± S.E.M. using GraphPad Prism. Each open circle represents the value of normalized log_10_(mean) for a target site. Each center line indicates the mean. Statistical significance was calculated using the two-tailed *t*-test. ^*^P < 0.05; ^***^P < 0.001. (C) The indicated cell lines transiently expressing different CRISPRa systems targeting *SOX2*, *OCT4*, or *HBB* were harvested for RT-qPCR analysis at 48 h post-transfection. *SOX2*, −185 bp; *OCT4*, −283 bp; *HBB*, −79 bp. Ctrl, no gRNA. The data were graphed as mean ± S.E.M. and represent three biological repeats. Statistical significance was calculated using the two-tailed *t*-test. ns, not significant; ^*^P < 0.05; ^**^P < 0.01;^***^P < 0.001. (D) HEK293T cells transiently expressing different CRISPRa systems and targeting different regions of the *HBG* and *IL1R2* locus were collected 48 h after transfection for RT-qPCR analysis. *HBG*, −85, −173, and −294 bp from TSS. *IL1R2*, −83, −187, and −397 bp from TSS. Ctrl, no gRNA. The data were graphed as mean ± S.E.M and represent three biological repeats. Statistical significance was calculated using one-way ANOVA. ns., not significant; ^*^P < 0.05; ^**^P < 0.01; ^***^P < 0.001. (E) The mean fold activation of each target site was calculated based on data from (D) and the values of log_10_(mean) of all target sites from one gene (*HBG* or *IL1R2*) were normalized with a minimum value of zero and a maximum value of one and plotted respectively using GraphPad Prism. Each symbol represents the value of normalized log_10_(mean) for a target site.

The FUS IDR domain (a.a. 1–212) is the smallest of the domains/protein we tested (Fig. S1). When fused to dCas9-VPR, dCas9-VPRF was among the most active at nearly all the loci examined (Figs. 1B–D and S2). Our studies are consistent with the previous result that the N-terminal QGSY-rich region (a.a. 1–165) of FUS (FUS IDR) is capable of promoting TF-mediated transcriptional activation by phase separation (Yang et al., 2014; Ryan et al., 2019; Owen et al., 2021). And the result showed that the fusion protein processed higher activation efficiency when FUS IDR fused to the C-terminus of dCas9-VPR (dCas9-VPRF) than to the N-terminus (F-dCas9-VPR) (Fig. S6a) and the C-terminus fusion protein was highly dynamic in cells (Fig. S6b), so we used the C-terminal fusion protein in the following experiment. To examine dCas9-VPRF activity further, we designed gRNAs to target both NTS and TS at different sites on different loci. Similar to dCas9-VPRN, dCas9-VPRF displayed both higher efficiency and a broader targeting window compared to dCas9-VPR (Figs. 4A, 4B and S7), although it did have a slight preference for the template strand. Furthermore, increased mRNA levels correlated with elevated protein expression as well in both HEK293T and K562 cell lines (Fig. S8).

Compared to the modified CRISPRa systems, dCas9-VPRF performed equally well at the *SOX2* and *HBB* target sites and performed significantly better at the *OCT4* target site (Fig. 4C). These results indicated that the three modified CRISPRa systems tested in our experiments could work well at some target sites but poorly performed at the others as previous reported (Tanenbaum et al., 2014; Konermann et al., 2015; Zhou et al., 2018). On the contrary, the dCas9-VPRF system worked well at different target sites with less preference. Notably, the activation efficiency of the three modified CRISPRa systems was efficient when targeting regions ~0 to 200 bp upstream of the TSS, but decreased rapidly ≥200 bp, which was consistent with previous studies (Gilbert et al., 2014; Konermann et al., 2015; Martella et al., 2019) (Figs. 4D, 4E and S9). In comparison, the dCas9-VPRF system continued to significantly activate gene transcription ≥200 bp upstream of the TSS. Taken together, our data demonstrate the ease and efficiency with which the dCas9-VPRF system can be used to activate endogenous gene expression at levels much higher than the SAM, SUN, and SPH CRISPRa systems.

### dCas9-VPRF mediates highly specific gene transcriptional activation

**Figure 5.**
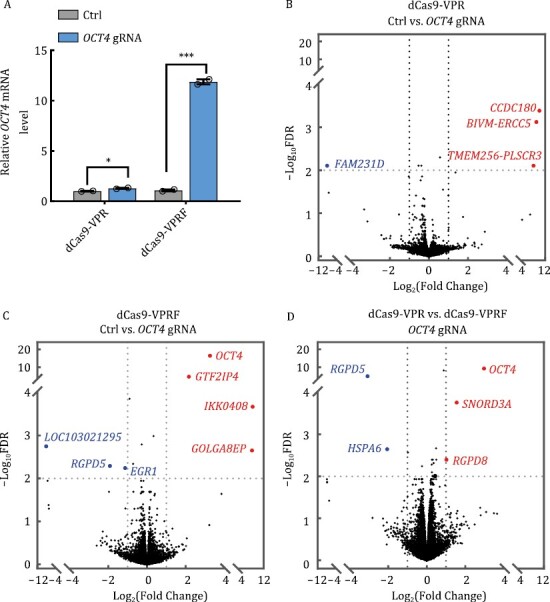
**The dCas9-VPRF CRISPRa system shows high specificity**. (A) HEK293T cells transiently expressing dCas9-VPR or dCas9-VPRF together with *OCT4*-targeting gRNAs (−103 bp from TSS) were harvested 48 h post-transfection for RT-qPCR analysis. Ctrl, no gRNA. The data were graphed as mean ± S.E.M. and represent two biological repeats. Statistical significance was calculated using a two-tailed *t*-test. ^*^P < 0.05; ^***^P < 0.001. (B and C) Cells from (A) were harvested for RNA-seq analysis (2 × 150 paired-end). The volcano map was plotted using GraphPad Prism. Genes in blue and red represent those that were down or upregulated respectively. Ctrl, no gRNA. (D) Data from (B) and (C) were processed using the Fastp and quantified based on the human transcriptome (hg38) by Salmon. Differential expression analyses were then performed using *R*. Genes with >2× fold change and <0.01 adjusted *P* values were considered significant (see Supplementary Methods for detailed descriptions). The data represent two independent biological repeats. FDR, false discovery rate.

Given the role of FUS in transcriptional regulation, we wanted to determine the impact of dCas9-VPRF expression and the extent of off-targets from fusing FUS IDR to dCas9-VPR. To this end, we first co-transfected into HEK293T cells an *OCT4*-targeting gRNA along with dCas9-VPRF or dCas9-VPR expression plasmids. As shown in Fig. 5A, RT-qPCR analysis indicates that dCas9-VPR alone could mildly induce *OCT4* expression by 1.3 folds, but dCas9-VPRF was able to upregulate *OCT4* expression by >10 fold. Next, we performed mRNA sequencing using both cell lines. RNA-seq results from two biological replicates for both cell lines were in good agreement with each other, indicating high reproducibility (Fig. S10). In line with the RT-qPCR results, the mRNA sequencing result showed that dCas9-VPRF significantly enhanced *OCT4* expression (8 folds vs. no gRNA control), as opposed to an undetectable activation of *OCT4* for dCas9-VPR alone (Fig. 5B and 5C). It has been shown that dCas9-VPR could efficiently activate *HBB* expression in HEK293T with high specificity and very few off-target effects (Dominguez et al., 2022). Consistently, both CRISPRa systems (dCas9-VPR and dCas9-VPRF) tested in this study had few off-target sites compared to the control group (Fig. 5B and 5C). For dCas9-VPR, three genes were upregulated and one was downregulated (Figs. 5B and S11). For dCas9-VPRF, three genes other than *OCT4* were upregulated and three other genes were downregulated (Figs. 5C and S11). Between the dCas9-VPR and dCas9-VPRF groups, only four genes excluding *OCT4* were differentially expressed which might be caused by FUS IDR overexpression [log_2_(Fold Change) >1 or <−1, FDR < 0.01] (Figs. 5D and S11), suggesting low off-target effects from FUS IDR fusion. Collectively, our findings suggest that dCas9-VPRF CRISPRa was able to specifically activate gene expression with minimal off-target activity.

## Discussion

In this study, we took advantage of phase separation to enhance CRISPRa-mediated gene expression control. Despite being implicated in diverse biological processes, all of the phase-separation proteins we tested were able to upregulate dCas9-VPR activity. The ability of these proteins to form multivalent condensate must be key to the process, where oligomerization of IDR-rich proteins through multivalent interaction can increase the concentration of dCas9-VPR at target sites, resulting in higher gene transcription activation efficiency. But the mechanisms underlying how IDR-rich proteins increase CRISPRa efficiency may be manifold. For instance, NUP98N and FUS IDR themselves have proven capable of recruiting TFs and epigenetic modification factors (Bai et al., 2006; Raczynska et al., 2015). The FG repeat of NUP98 can recruit EP300 to activate gene transcription (Bai et al., 2006). FUS can activate gene transcription by interacting with U7 snRNPs and histone-specific TFs (Raczynska et al., 2015). NUP98N and FUS IDR in the dCas9-VPRN and dCas9-VPRF fusion proteins may be able to recruit TFs and epigenetic modifiers in addition to increasing dCas9-VPR. Such possibilities warrant further investigation. One concern with this approach is possible non-specific gene activation. However, the enhancement of transcriptional activation by the IDR domains was clearly dependent on VPR. Furthermore, our analysis indicates that the specificity of the dCas9-VPRF system was comparable to the dCas9-VPR system. Our findings support the use of phase-separation proteins/domains as a viable and effective means to enhance CRISPRa activity.

Phase-separation mediated multivalent interaction also plays an essential role in gene silencing. For example, the IDR-rich CBX subunit of the polycomb repressive complex cPRC1 can recognize histone H3K27me3 modifications and induce cPRC1 condensation to compact chromatin, which can lead to gene expression silencing (Guo et al., 2021). HP1 droplet formation physically sequesters and compresses chromatin, enabling repressive factor recruitment (Larson et al., 2017). It will be interesting to explore whether multivalent condensates can similarly inhibit gene expression through enhancing CRISPRi activity.

Compared with dCas9-VPR, both the dCas9-VPRN and dCas9-VPRF systems showed broader gRNA-targeting windows and reduced DNA strand preference. Having targeting windows that extend further upstream of the TSS should help minimize interference with the recruitment of other factors to *cis*-regulatory elements near the TSS. And less strand bias may increase the number of effective gRNAs. Such characteristics could make dCas9-VPRN and dCas9-VPRF attractive alternatives to canonical and several other modified CRISPRa systems. If vector size is not a limiting factor, both dCas9-VPRN and dCas9-VPRF may be used to activate endogenous genes. The smallest size dCas9-VPRF showed the highest transcriptional activation efficiency at most of the target sites examined compared to other well-known CRISPRa systems (e.g., SAM, SUN, and SPH). In addition, it is small and requires only two components, which should make for easier delivery into cells/tissues. It is conceivable that if SpCas9 is further replaced with smaller Cas proteins such as IscB (Kapitonov et al., 2015; Altae-Tran et al., 2021; Schuler et al., 2022) and Cas12f (Xu et al., 2021b), the resulting fusion proteins along with the gRNA may be packaged into a single vector (e.g., adeno-associated viral vectors) that may achieve even higher *in vivo* delivery efficiency. It is also worthwhile to investigate whether one or partial VPR component could be omitted, thus reducing the size of VPRF itself while maintaining its activity. By fusing to the FUS IDR domain with some optimization related to CRISPR-Cas protein and TFs (e.g., Cas9 protein homologs and TFs simplification), the CRISPRa system may achieve single AAV packaging as well as high activity.

Stable long-term gene activation may be required to cure genetic diseases *in vivo*. For example, to treat β-thalassemia, activated *HBG* expression will need to be maintained in HSC. Whether the dCas9-VPRN and dCas9-VPRF CRISPRa systems can facilitate epigenetic modifications and activate gene expression in a long-term manner may need to be investigated in future studies. However, prolonged expression and phase separation of dCas9-VPRN and dCas9-VPRF may lead to other side effects, for example, the immunogenicity of Cas9 nuclease (Charlesworth et al., 2019; Wagner et al., 2019), off-target activation (Tsai and Joung, 2016), and liquid-solid phase transition of phase-separation condensates (Patel et al., 2015; Schmidt and Görlich, 2015; Ibáñez de Opakua et al., 2022). Alternatively, conditional expression strategies could be applied to minimize these effects. In addition to applications in basic research, transient activation of endogenous genes by dCas9-VPRN or dCas9-VPRF could be used in clinical applications when upregulated genes including transcription regulators result in cell fate changes such as differentiation (Trapnell et al., 2014; Tsankov et al., 2015). To achieve such long-term effects, additional transcription co-activators and epigenetic factors can be combined with the IDR domains. The dCas9-VPRN and dCas9-VPRF CRISPRa systems may be ideal candidates for this area of investigation.

## Materials and methods

### Cell line, antibodies, and other procedures

HEK293T, HeLa, and K562 cells were obtained from ATCC and cultured in Dulbecco’s modified Eagle medium (Corning) (HEK293T and HeLa) or RPMI 1640 medium (K562) supplemented with 10% fetal bovine serum (ExCell) respectively. All the cells were at 37°C and 5% CO_2_ in a humidified incubator and mycoplasma negative. For transient transfection. Cells were either transfected using the PEI (Polysciences) or LipoFectMax 3000 (ABP Biosciences) following the manufacturer’s instructions or electroplated using the Nucleofector system (program FF-120). For the latter, approximately 1 × 10^6^ of K562 cells were centrifuged at 90×*g* for 10 min, resuspended in 20 μL of nucleofection solution, and then mixed with 2.5 μg plasmid DNA. The mixtures were loaded into a 16-well nucleocuvette strip (Lonza) for nucleofection (Lonza) as described before with optimization (Hendel et al., 2015; Koblan et al., 2021). The molar ratio of the dCas9-fusion protein to gRNA was approximately 2꞉3 in all the experiments without specific mention. Specifically, in the experiment of dCas9-VPRN MUT gene activation in Fig. 2F, the same amount of mass of total plasmids was co-transfected, and the molar ratio of dCas9-VPR or dCas9-VPRN to gRNA was 2꞉3, whereas that of dCas9-VPRN MUT to gRNA was 1꞉3 with pUC19 supplemented the rest mass. For live-cell imaging, cells were seeded on 35 mm confocal petri dishes (Hanning78416) 12 h before transfection, and images were taken 24 h after transfection. For 1,6-hexanediol treatment (1,6-HD), cells were imaged immediately before 1,6-HD addition (5%) and then were imaged every 2 s for 2 min. All images were captured on a LEICA TCS-SP5 laser scanning confocal microscope with a 488 nm laser. Western blot analysis was performed as previously described (Chen et al., 2020). The antibodies used in this study are, rabbit polyclonal anti-NAP1L1 antibody (Proteintech) (1:1000 dilution), rabbit polyclonal anti-Hemoglobin subunit gamma 1 and 2 antibody (HUABIO) (1:2,000 dilution), rabbit polyclonal anti-GAPDH antibody (Abmart) (1:1,000 dilution), rabbit polyclonal anti-H3 antibody (Abcam), goat anti-rabbit secondary antibody (Odyssey) (1:5,000 dilution), and goat anti-mouse secondary antibody (Odyssey) (1:5,000 dilution).

### Vectors

dCas9-VPR was PCR amplified and sub-cloned into pX601 (Addgene, #61591) to generate the pXdCas9-VPR vector (Chen et al., 2020), which was used for cloning the NUP98N fragment (a.a. 1–514) (either 5ʹ of dCas9 or 3ʹ of VPR). Monomeric super-folder GFP was then ligated into the above two constructs to obtain pxGFP-dCas9-VPR-NUP98N and pxNUP98N-dCas9-VPR-GFP. Other phase-separation protein sequences or their IDR region, including FUS IDR (a.a. 1–212), FCA PrLD (a.a. 349–747), ARID3B (a.a. 1–567), COIL (a.a. 1–576), NCL (a.a. 1–710), NIFK (a.a. 1–293), NPM1 (a.a. 1–294), NUP50 (a.a. 1–468), SMN1 (a.a. 1–294), SRSF2 (a.a. 1–221), PSPC1 (a.a. 1–523) and NUP98N MUT (Ahn et al., 2021) were similarly sub-cloned into the pxGFP-dCas9-VPR-NUP98N vector (replacing the NUP98N sequence). The expression plasmids for the SAM, SUN, and SPH systems were gifts from Hui Yang’s lab (Zhou et al., 2018). gRNA sequences were cloned into the lenti-Guide-Puro vector (Addgene, #52963). All the gRNA and protein sequences are listed in Tables S1 and S2, respectively.

### Fluorescence recovery after photobleaching (FRAP) assays

Cells (2.5 × 10^5^) were seeded on 35 mm confocal petri dishes (Hanning78416) and assayed 24 h after transfection on a LEICA TCS-SP5 laser scanning confocal microscope with a 488 nm laser. Images were first acquired with the 488 nm laser intensity at <10% power to preserve overall signals. The laser intensity was increased to 100% power for photobleaching in an area of ~1 μm diameter. Immediately after the photobleaching, images were collected every second on one Z-stack for 100 images. The fluorescent intensity of the condensates was measured, and background signals were subtracted. For each group, 15 cells with no less than one condensate per cell were bleached. The recovery curve was analyzed and fitted to the *One phase decay* function with GraphPad Prism 9. The half-life values (*T*_1/2_) were determined by the software after fitting.

### Quantitative reverse transcription polymerase chain reaction (RT-qPCR)

qPCR was performed as described previously (Chen et al., 2020). Briefly, total RNA was extracted using the TRIzol reagent (TAKARA) and quantified by Nanodrop 1000. cDNA was prepared using the cDNA reverse transcription kit (TAKARA). Approximately 1,000 ng of total RNA was used for each reverse transcription reaction. Real-time PCR was performed with the qTOWER^3^ system (Analytikjena) using the 2× Color SYBR Green qPCR Master Mix (EZBioscience). Relative mRNA levels were determined and analyzed using the −∆∆Ct method. All primers are listed in Table S3.

### RNA-seq and data analysis

Cells were collected two days after transfection for RNA extraction and reverse transcription as described above. RNA-seq (2 × 150 paired-end) was performed according to the standard Illumina protocols by Azenta Life Science (Suzhou, China). For library preparation, 1 µg of total RNA per sample was used as input. Sequencing libraries were generated using the Next^®^ Ultra RNA Library Prep Kit for Illumina^®^ (NEB) following the manufacturer’s protocol. Each sample was PCR amplified using P5 and P7 primers. The libraries were analyzed on the Illumina Novaseq instrument. The Fastp (version 0.20.0) was applied to filter out low-quality reads, cut adapters, and check the quality of raw FASTQ files to obtain clean reads (Chen et al., 2018). Clean reads were then used to quantify gene expression levels based on the human transcriptome (hg38) (downloaded from AWS iGenomes, derived from Illumina iGenomes) by Salmon (version: 1.8.0) (Patro et al., 2017). Differential expression analyses were performed in the R software (version: 4.1.3). Specifically, gene abundance matrices were imported and summarized from the Salmon results via R package tximport (version: 1.22.0) (Soneson et al., 2015). Differentially expressed genes were determined by the quasi-likelihood *F*-test method in edgeR (version: 3.36.0) (Robinson et al., 2010). A > 2 folds change and a < 0.01 adjust-*P* value was selected as the significance cutoffs. Detailed information of the raw counts and off-target genes is listed in Tables S4 and S5.

### Statistical analysis

Statistical analyses were carried out with GraphPad Prism 9. Error bars represent the standard error of the mean (S.E.M.) and results were presented as mean ± SEM. One-way ANOVA or unpaired two-tailed *t*-tests were used to calculate *P* values. Detailed information of the off-target genes is listed in Table S4.

## Supplementary Material

pwad013_suppl_Supplementary_Materials

pwad013_suppl_Supplementary_Table_S1

pwad013_suppl_Supplementary_Table_S2

pwad013_suppl_Supplementary_Table_S3

pwad013_suppl_Supplementary_Table_S4

pwad013_suppl_Supplementary_Table_S5

## Data Availability

All relevant data and materials are available upon request. Sequences of plasmids for expression of phase-separation proteins or IDR domains, including NUP98N (human), FUS IDR (human), FCA PrLD (*Arabidopsis*), ARID3B (mouse), COIL (human), NCL (human), NIFK (human), NPM1 (human), NUP50 (human), SRSF2 (human), SMN1 (human), PSPC1 (human) and NUP98N mutant are listed in Table S2. Raw FASTQ files of the RNA-seq data have been deposited in the NCBI database and are available at NCBI BioProject: PRJNA901619.

## Abbreviations

ARID3B: AT-rich interactive domain-containing protein 3B
CRISPR: clustered regularly interspaced short palindromic repeats
COIL: the cajal body component COIL
CRISPRa: CRISPR activation
FCA: flowering time control protein FCA
FUS: fused in sarcoma
*HBB*: hemoglobin subunit beta
*HBG*: hemoglobin subunit gamma 1 and 2
IDR: intrinsically disordered regions
*IL1B*: interleukin 1 beta
*IL1R2*: interleukin 1 receptor type 2
*IL1RN*: interleukin 1 receptor antagonist protein
*NAP1L1*: nucleosome assembly protein 1 like 1
NCL: nucleolin
NIFK: nucleolar protein interacting with the FHA domain of MKI67
NPM1: nucleophosmin
NUP50: nucleoporin 50
NUP98: nucleoporin 98 proteins
*OCT4*: also named
*POU5F1*: POU class 5 homeobox 1
PSPC1: paraspeckle protein component 1
SMN1: survival of motor neuron 1
*SOX2*: SRY-box transcription factor 2
SRSF2: splicing factor, arginine/serine-rich 2
VPR: VP64-P65-RTA.
