# Supplementary material for: CRISPR-assisted transcription activation by phase-separation proteins
Source: Protein Cell. 2023 Mar 11;14(12):874–87. doi: 10.1093/procel/pwad013 (PMC10691850; doi:10.1093/procel/pwad013)
Supplement: pwad013_suppl_Supplementary_Materials [file pwad013_suppl_supplementary_materials.pdf]

Supplementary Figure 1

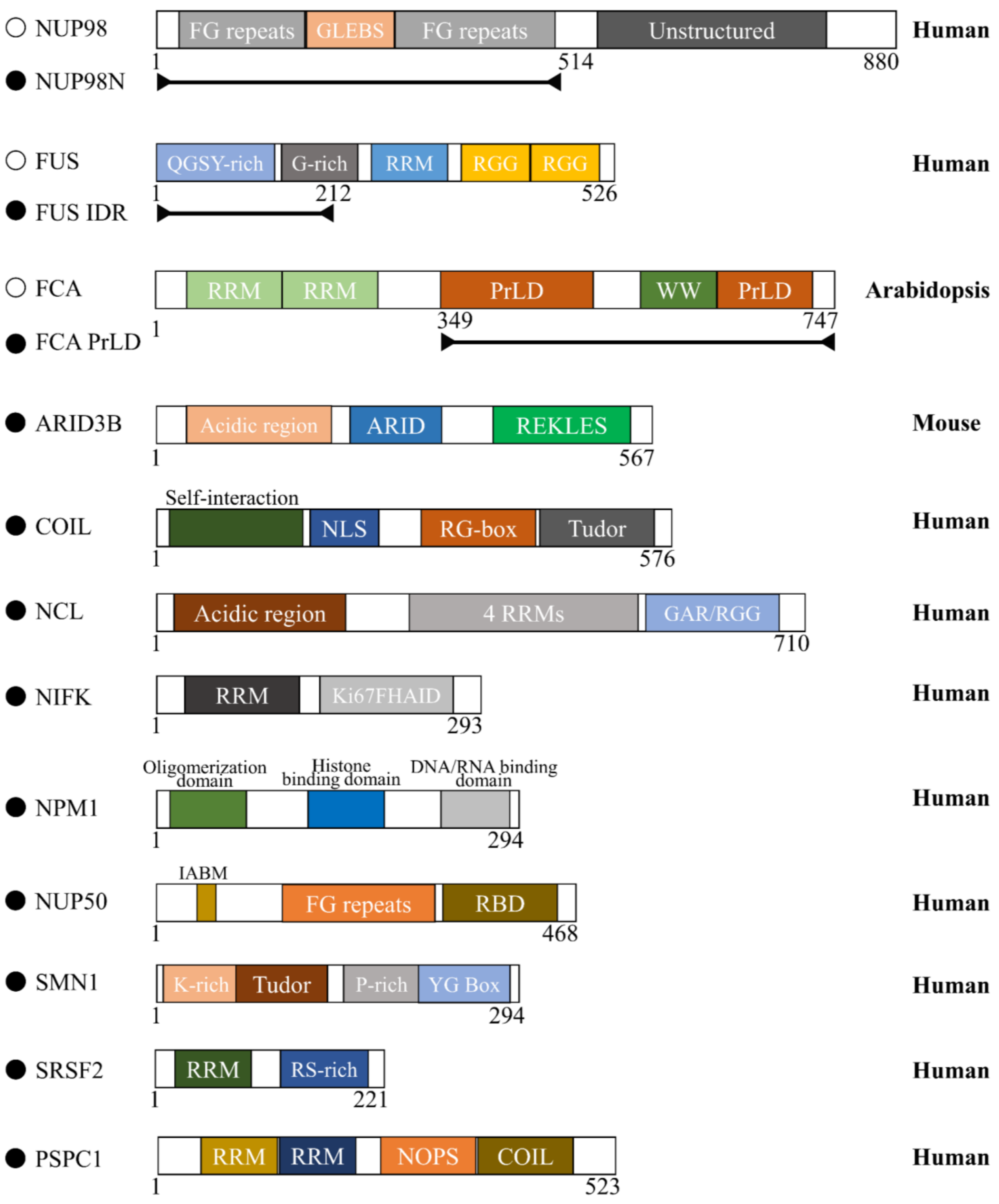

Supplementary Figure 1. An illustration of the domain structures of the phase-separation proteins tested in our study.

FG repeats, phenylalanine-glycine (FG) repeats, a motif commonly found in nucleoporins. GLEBS, a Gle2-binding-sequence domain. QGSY-rich, glutamine, glycine, serine, and tyrosine-rich region. G-rich, glycine rich region. RRM, RNA recognition motif. RGG, arginine and glycine-rich region with RGG repeats. PrLD, prion-like domain with prion-like amino acid composition. WW, also called WWP, is a domain that contains two highly conserved residues, tryptophan (W) and proline (P). ARID, AT-rich interactive domain. REKLES, a conserved amino acid motif. NLS, nuclear localization signal. RG-box, a region rich in arginine (R) and glycine (G). Tudor, the Tudor domains. 4 RRMs, is a domain that contains four RNA-recognition motifs (RRMs). GAR/RGG, glycine and arginine-rich (GAR) or arginine-glycine-glycine repeats (RGG) domain. Ki67FHAID, Ki67-FHA (forkhead associated (FHA) domain of Ki67) interaction domain. IABM, importin-α binding motif. RBD, Ran-binding domain. RS-rich, arginine/serine-rich domain. K-rich, lysine-rich region. P-rich, proline-rich region. YG-box, a highly conserved oligomerization domain that contains a $(YxxG)_3$ motif. NOPS, NonA/paraspeckle domain. COIL, coiled-coil domain.

Supplementary Figure 2

a

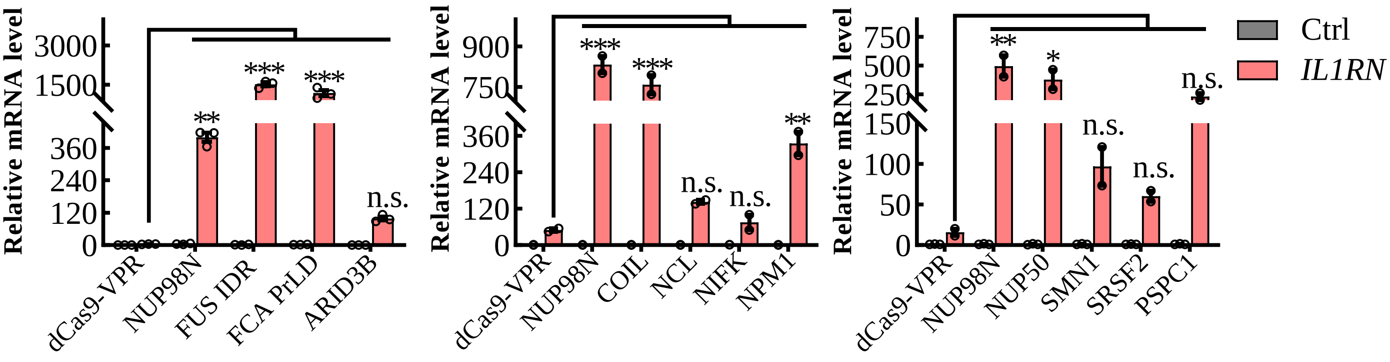

b

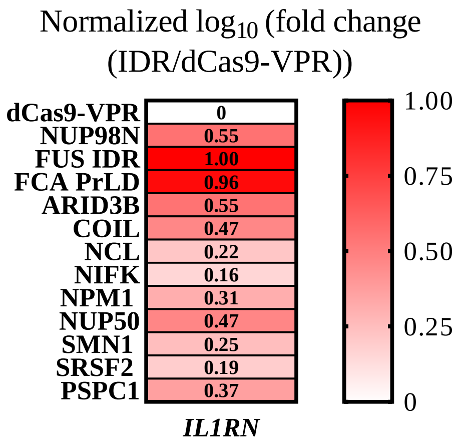

Supplementary Figure 2. Screening of IDR-rich proteins by fusing to dCas9-VPR for efficient CRISPRa systems.
(a) HEK293T cells transiently expressing dCas9-VPR fused to the indicated phase-separation proteins/domains (C-terminal fusion) together with an *IL1RN*-targeting gRNA (-9 bp of TSS) were harvested for RT-qPCR analysis of mRNA expression 48 hrs after transfection. Ctrl, no gRNA. The results from three biological repeats are graphed as mean ± S.E.M. Statistical significance was calculated using one-way ANOVA. ns, not significant; *, $p < 0.05$; **, $p < 0.01$; ***, $p < 0.001$.
(b) Data from (a) were processed to obtain the ratio for fold changes of the indicated IDR fusion proteins over dCas9-VPR alone. Normalized $\log_{10}$ (fold change) with a minimum value of zero and a maximum value of one was obtained by GraphPad Prism and displayed in the heat map.

Supplementary Figure 3

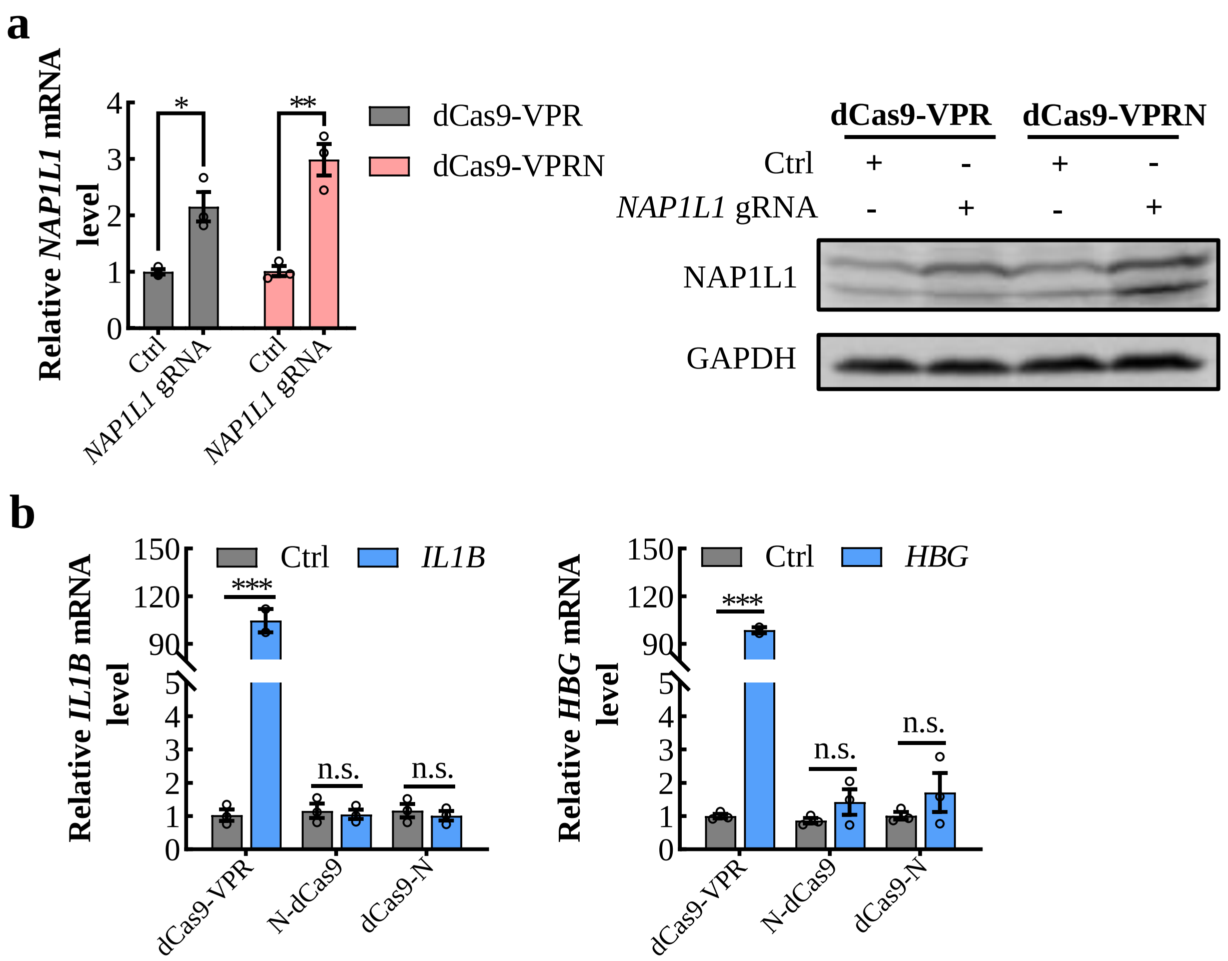

Supplementary Figure 3. dCas9-VPRN enables more efficient CRISPRa activity in a VPR-dependent manner.

(a) HEK293T cells transiently expressing dCas9-VPR or dCas9-VPRN together with a pool of *NAP1L1*-targeting gRNAs targeting different regions upstream of the TSS (-53, -87, -189, -286, -366, and -479 bp) were collected at 48 hours post-transfection for RT-qPCR (left) and western blot analysis (right). Ctrl, no gRNA. The RT-qPCR data from three biological repeats are presented as mean ± S.E.M. Statistical significance was calculated using two-tailed t-tests. *, $p < 0.05$; **, $p < 0.01$.

(b) HEK293T cells transiently expressing dCas9-VPR, N-dCas9, or dCas9-N with gRNA (targeting *IL1B* or *HBG*) were harvested 48 hours post-transfection. N-dCas9, NUP98N-dCas9. dCas9-N, dCas9-NUP98N. Ctrl: no gRNA. The data of three biological repeats were displayed as mean ± S.E.M. Statistical significance was calculated using two-tailed t-tests. n.s., not significant; ***, $p < 0.001$.

Supplementary Figure 4

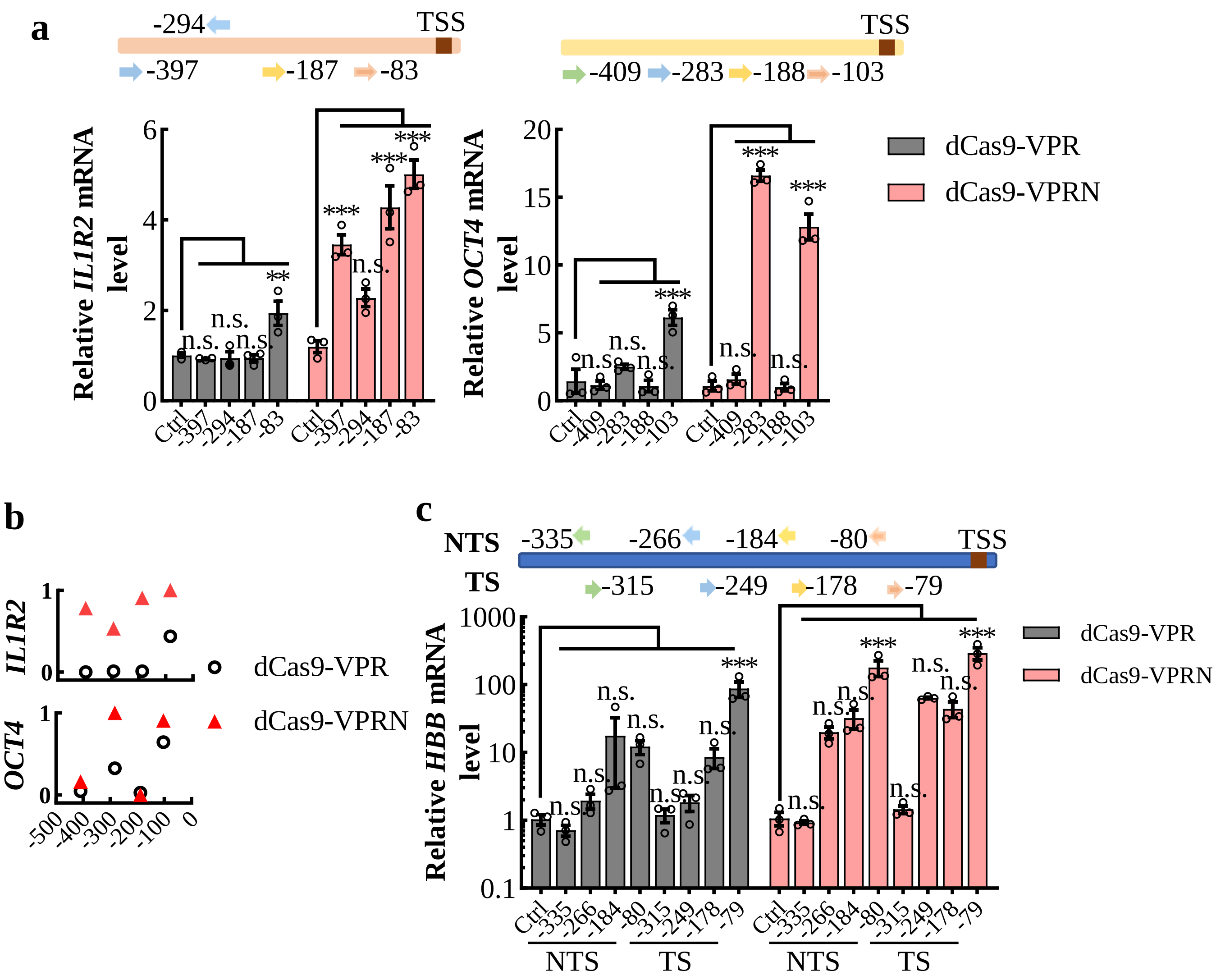

Supplementary Figure 4. Characteristics of dCas9-VPRN CRISPRa system.

(a) HEK293T cells transiently expressing dCas9-VPR or dCas9-VPRN with gRNAs targeting the regions upstream of the TSS of *IL1R2* or *OCT4* (top, arrows represent the specific positions) were harvested 48 hours post-transfection for mRNA level detection (bottom). Ctrl: no gRNA. Results from three biological repeats were graphed as mean ± S.E.M. Statistical significance was calculated using one-way ANOVA. n.s., not significant; **, $p < 0.01$; ***, $p < 0.001$.

(b) The mean fold activation was calculated based on data from (a) and the values of $\log_{10}$ (mean) from each gene were normalized with a minimum value of zero and a maximum value of one and plotted with GraphPad Prism. Each open circle or triangle represents normalized $\log_{10}$ (mean) values of target sites.

(c) HEK293T cells transiently co-transfected with dCas9-VPR or dCas9-VPRN expression plasmids along with gRNAs targeting the indicated sites (top) were harvested 48 hours after transfection for RT-qPCR analysis (bottom). NTS, non-template strand; TS, template strand. Ctrl: no gRNA. Data from three biological repeats were shown as mean ± S.E.M. Statistical significance was calculated using one-way ANOVA. n.s., not significant; ***, $p < 0.001$.

Supplementary Figure 5

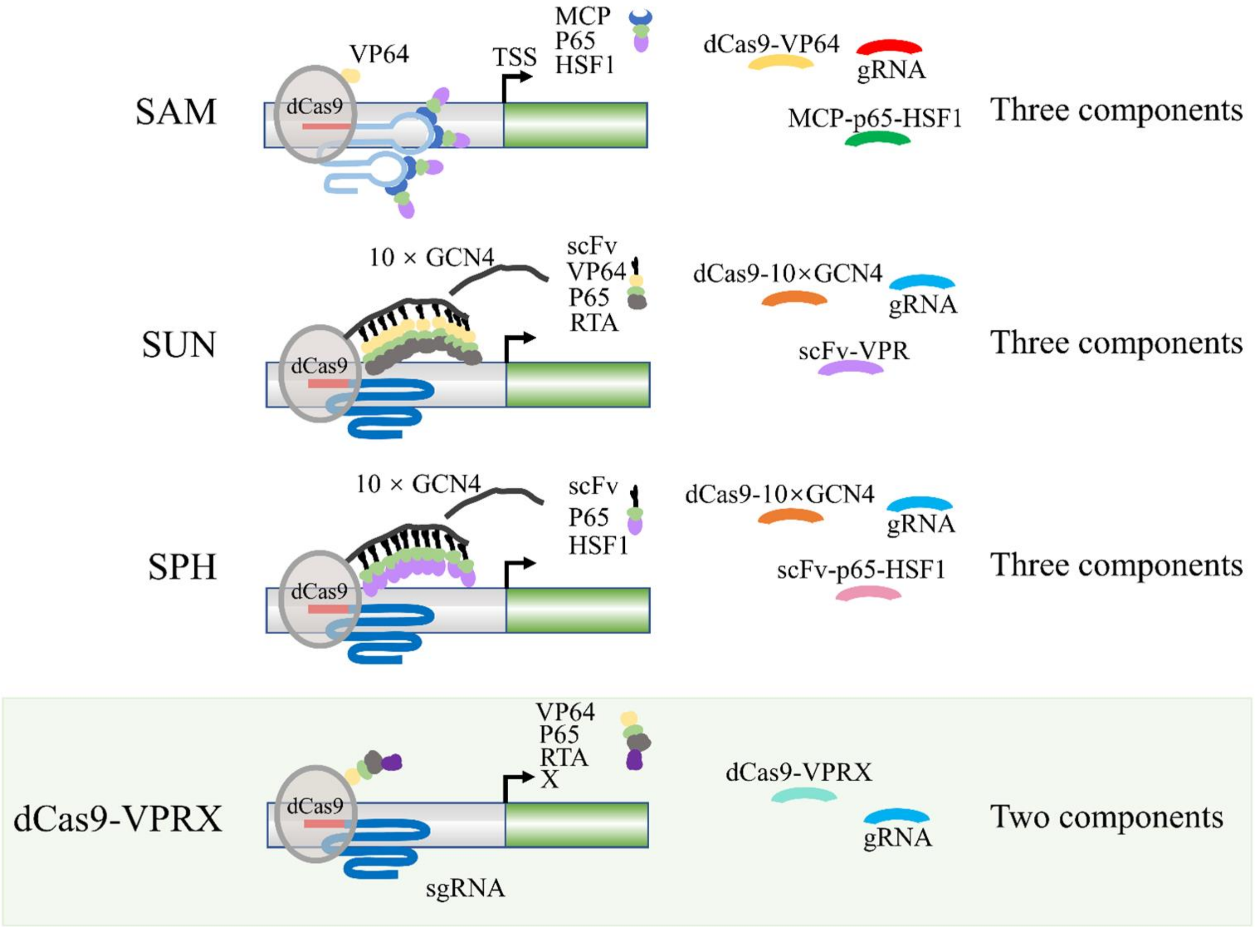

Supplementary Figure 5. An illustration of the CRISPRa systems tested in our study.
SAM CRISPRa uses three separate vectors that encode dCas9-VP64, MCP-P65-HSF1, and the MS2-containing chimeric gRNA. SUN and SPH CRISPRa systems use three separate vectors that encode the gRNA, dCas9-10×GCN4, and a transcription factor (TF), where scFv-VP64-P65-Rta and scFv-P65-HSF1 serve as the TF for SUN and SPH respectively. In comparison, the dCas9-VPRX CRISPRa system being developed here relies on two expression vectors encoding the gRNA and the fusion protein between dCas9-VPR and phase-separation protein domains.

Supplementary Figure 6

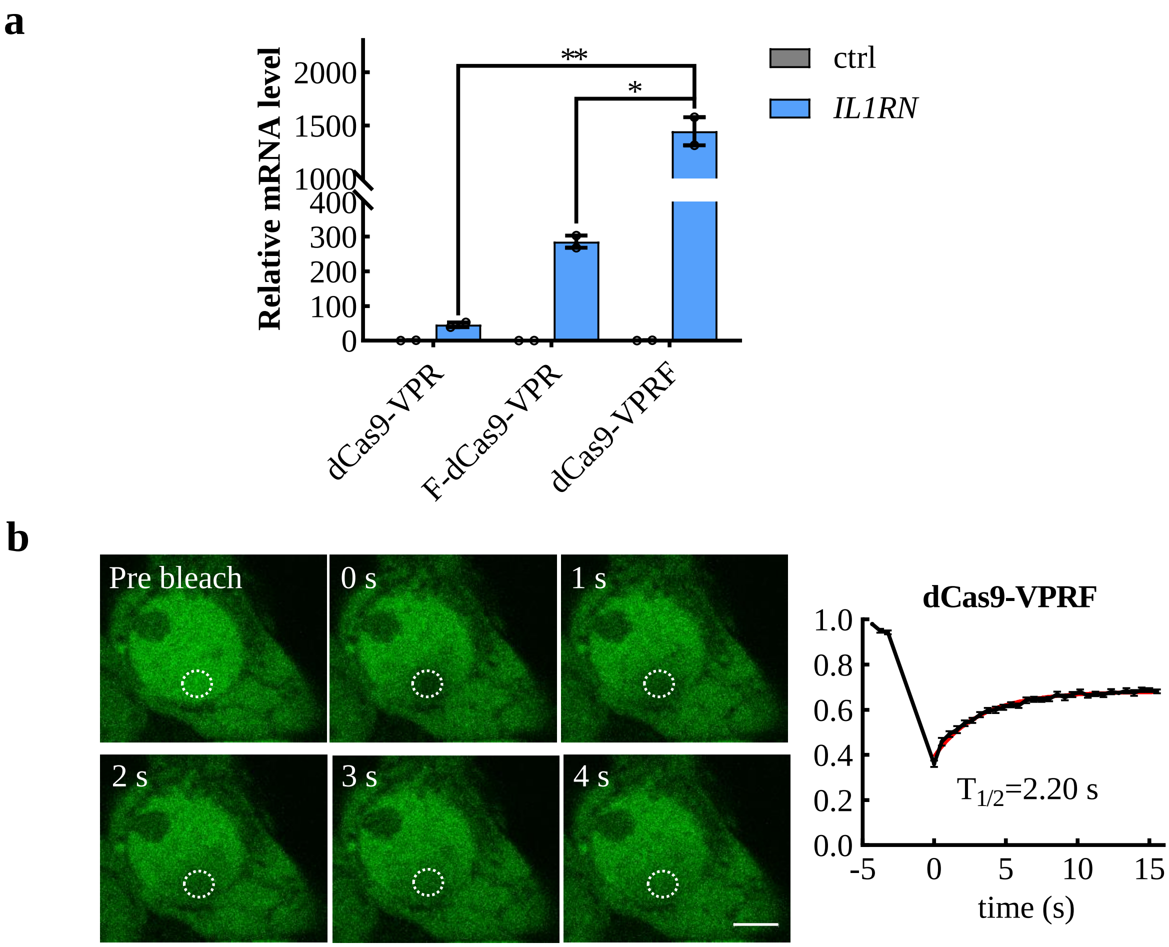

Supplementary Figure 6. The dCas9-VPR-FUS IDR fusion protein enable efficient CRISPRa activity and is highly dynamic.

(a) HEK293T cells transiently expressing dCas9-VPR, F-dCas9-VPR, or dCas9-VPRF with gRNA (targeting *IL1RN*) were harvested 48 hours post-transfection. F-dCas9-VPR, FUS IDR-dCas9-VPR. dCas9-VPRF, dCas9-VPR-FUS IDR. Ctrl: no gRNA. The data of three biological repeats were displayed as mean ± S.E.M. Statistical significance was calculated using two-tailed t-tests. *, $p < 0.05$; **, $p < 0.01$.

(b) Live cell imaging and fluorescence recovery after photobleaching (FRAP) analysis of the HEK293T cells 24 hours after transient transfection of the dCas9-VPRF protein. Scale bar, 5 μm. Left, representative images of cells at the indicated time points following photobleaching. Right, the recovery curve of the bleaching points was plotted mean ± S.E.M. Three independent experiments were performed with ~15 cells/experiment. All cells had at least one photobleached point. The recovery curve was fitted to determine the half-life (T1/2).

Supplementary Figure 7

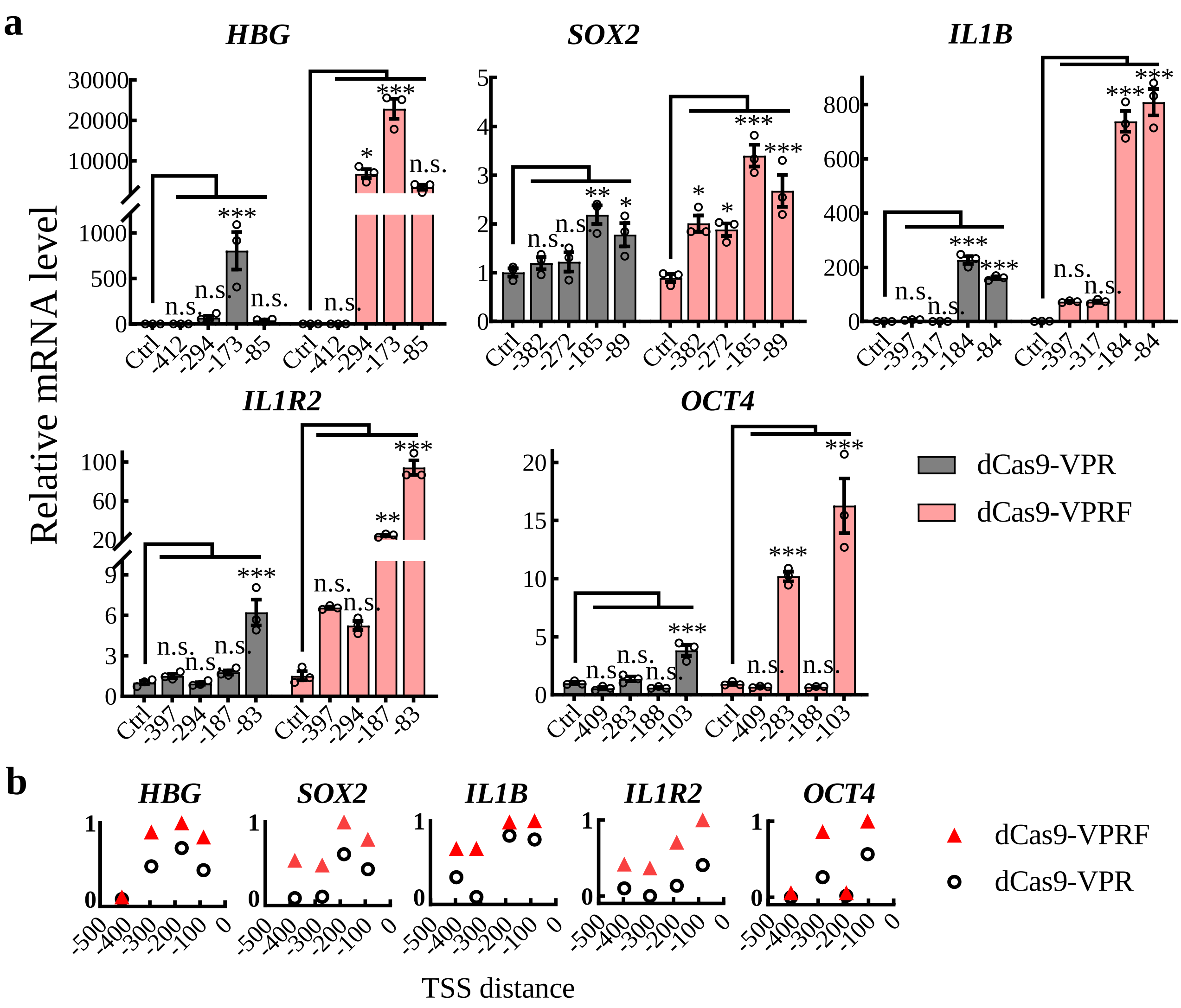

Supplementary Figure 7. Characteristics of dCas9-VPRF CRISPRa system

(a) HEK293T cells transiently expressing dCas9-VPR or dCas9-VPRF with gRNAs (same as Fig. 3a and Supplementary Fig. 4a) targeting the indicated regions upstream of the TSS of *HBG*, *SOX2*, *IL1B*, *IL1R2*, and *OCT4* were harvested 48 hours after transfection. Ctrl, no gRNA. Data from three biological repeats were plotted as mean ± S.E.M. Statistical significance was calculated using one-way ANOVA. n.s., not significant; *, $p < 0.05$; **, $p < 0.01$; ***, $p < 0.001$.

(b) The mean fold activation of each target site was calculated based on data in (a) and the $\log_{10}$(mean) values from each gene were normalized with a minimum value of zero and a maximum value of one and plotted using GraphPad Prism. Each open circle or triangle represents the value of normalized $\log_{10}$(mean) for a target site.

Supplementary Figure 8

a

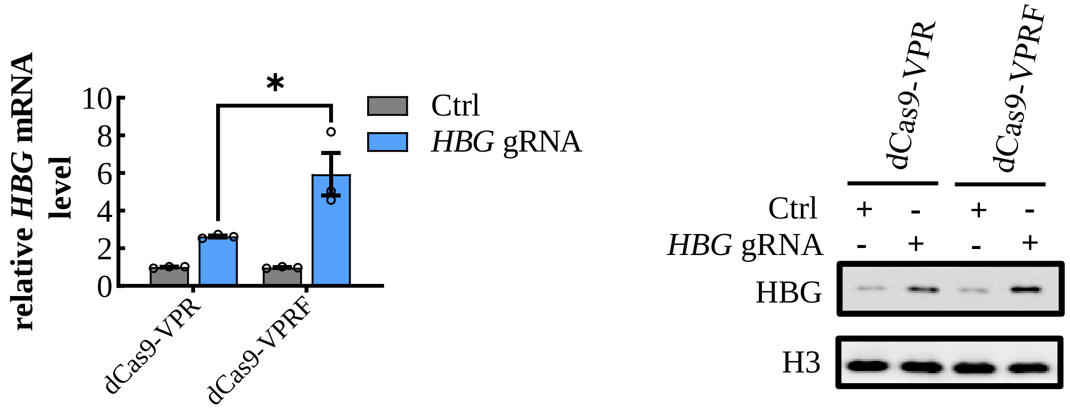

b

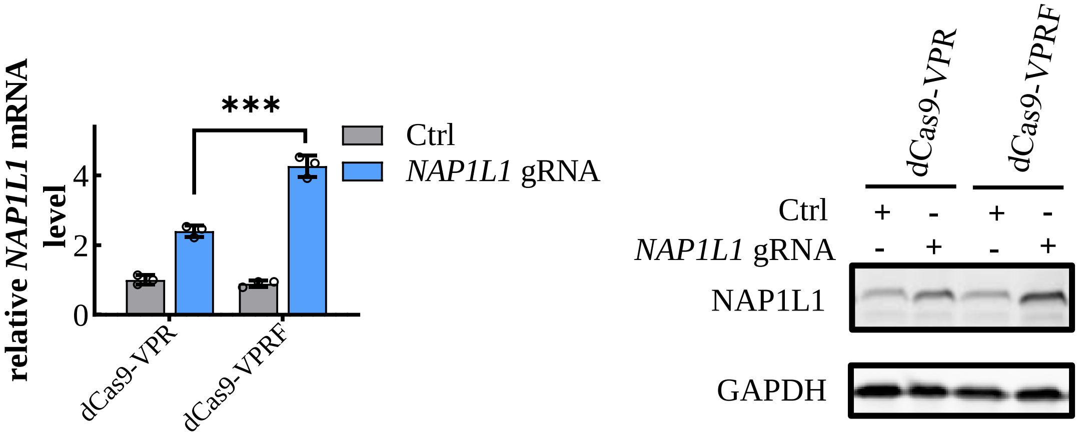

Supplementary Figure 8. Elevation of mRNA level by dCas9-VPRF system correlated with elevated protein expression.

(a) K562 cells transiently expressing dCas9-VPR or dCas9-VPRF together with a single gRNA targeting *HBG* (-173 bp from TSS) were collected at 48 hrs after transfection for RT-qPCR (left) and western blot analysis (right). Ctrl, no gRNA. The RT-qPCR data were graphed as mean ± S.E.M and represent three biological repeats. Statistical significance was calculated using the two-tailed t-test. *, $p < 0.05$.

(b) HEK293T cells transiently expressing dCas9-VPR or dCas9-VPRF together with a pool of six gRNAs targeting *NAP1L1* (-53, -87, -189, -286, -366, -479 bp from TSS) were collected at 48 hrs after transfection for RT-qPCR (left) and western blot analysis (right). Ctrl, no gRNA. The RT-qPCR data were graphed as mean ± S.E.M and represent three biological repeats. Statistical significance was calculated using the two-tailed t-test. ***, $p < 0.001$.

Supplementary Figure 9

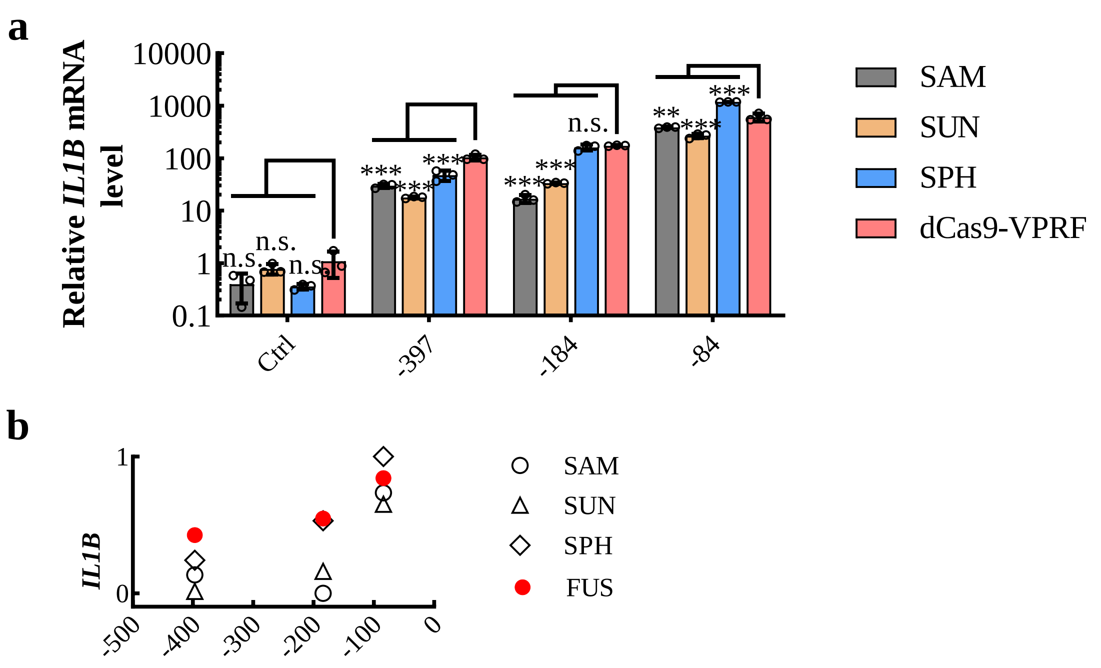

Supplementary Figure 9. The dCas9-VPRF system is efficient and less restrictive than other modified CRISPRa systems.

(a) HEK293T cells transiently expressing the indicated CRISPRa systems with a series of *IL1B*-targeting gRNAs (-84, -184, and -397bp upstream of the TSS) were harvested for RT-qPCR analysis 48 hrs after transfection. Ctrl, no gRNA. The data from three biological repeats were plotted as mean ± S.E.M. Statistical significance was calculated using one-way ANOVA. n.s., not significant; *, $p < 0.05$; **, $p < 0.01$; ***, $p < 0.001$.

(b) The mean fold activation of each target site was calculated based on data from (a) and the values of $\log_{10}$ (mean) were normalized with a minimum value of zero and a maximum value of one and plotted using GraphPad Prism. Each symbol represents the normalized $\log_{10}$ (mean) value.

Supplementary Figure 10

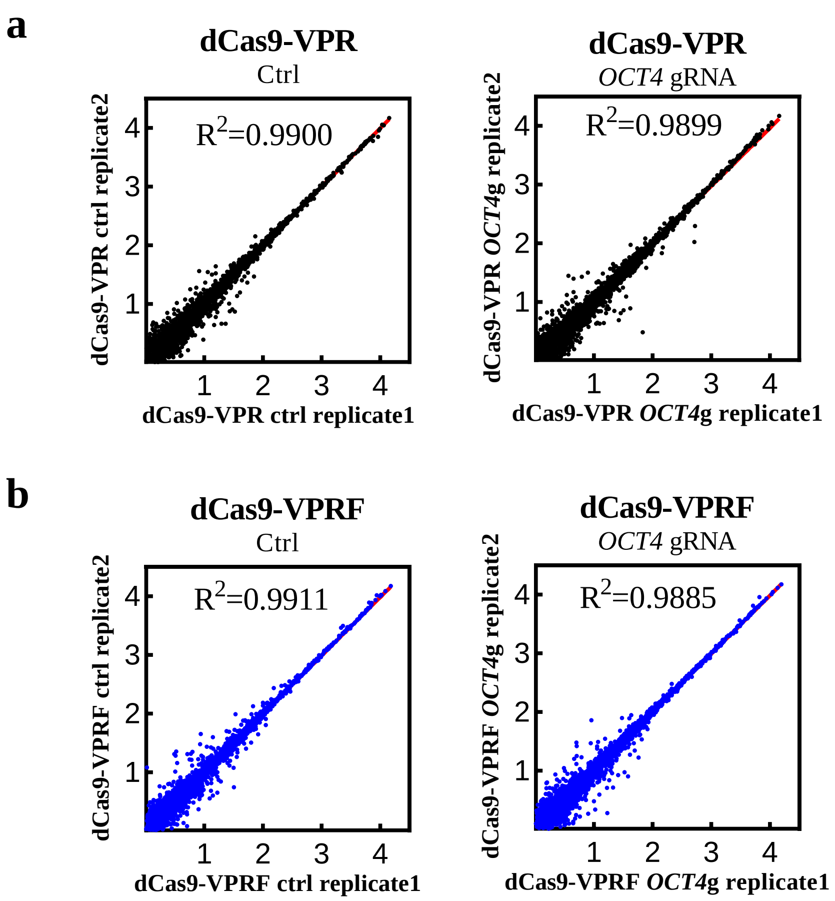

Supplementary Figure 10. The RNA-seq results in figure 5 are in high reproducibility.

(a-b) Scatterplots comparing the $\log_{10}$ (TPM+1) values of biological replicates of control vs. gRNA groups for dCas9-VPR (a) and dCas9-VPRF (b). Ctrl, no gRNA. *OCT4*, a gRNA targeting -103 bp of *OCT4* TSS. All data points were fitted to a simple linear regression model using GraphPad Prism and R^2 represents the correlation coefficient.

Supplementary Figure 11

dCas9-VPR				dCas9-VPR-FUS IDR				dCas9-VPR v.s. dCas9-VPRF			
gene name	log_2 FC	FDR	threshold	gene name	log_2 FC	FDR	threshold	gene name	log_2 FC	FDR	threshold
BIVM-ERCC5	**10.0570**	**0.0004**	**Up**	*OCT4*	**3.2607**	**3.22E-17**	**Up**	*OCT4*	**2.9757**	**5.18E-10**	**Up**
CCDC180	**9.0567**	**0.0008**	**Up**	*KIAA0408*	**8.5059**	**0.0002**	**Up**	*SNORD3A*	**1.5543**	**0.0002**	**Up**
TMEM256-PLSCR3	**8.0507**	**0.0078**	**Up**	*GOLGA8EP*	**8.2665**	**0.0022**	**Up**	*RGPD8*	**1.0276**	**0.0040**	**Up**
FAM231D	**-8.0974**	**0.0078**	**Down**	*GTF2IP4*	**2.1684**	**1.69E-05**	**Up**	*HSPA6*	**-2.0528**	**0.0023**	**Down**
				EGR1	**-1.1353**	**0.0058**	**Down**	*RGPD5*	**-3.0765**	**9.46E-06**	**Down**
				RGPD5	**-1.9212**	**0.0052**	**Down**				
				LOC103021295	**-8.0569**	**0.0018**	**Down**				

Supplementary Figure 11. The off-target gene lists of the RNA-seq data in figure 5.

Off-target genes identified by comparing dCas9-VPR *OCT4*-targeting v.s. dCas9-VPR ctrl (left), dCas9-VPRF *OCT4*-targeting v.s. dCas9-VPRF ctrl (middle), and dCas9-VPRF *OCT4*-targeting v.s. dCas9-VPR *OCT4*-targeting (right) are listed. For each comparison, the gene name, fold change of mRNA levels (log_2FC), false discovery rate (FDR), and type of change (threshold) are displayed. Detailed information is listed in Supplementary Table 4.
